# Exploring Saudi Physical Therapists' Perceptions and Opinions on Their Profession: A Mixed-Method Study

**DOI:** 10.1155/2022/2890548

**Published:** 2022-06-21

**Authors:** Saleh M. Aloraini, Ghdy R. Alrsheed

**Affiliations:** Department of Physical Therapy, College of Medical Rehabilitation, Qassim University, Saudi Arabia

## Abstract

**Methods:**

A cross-sectional, mixed-method study was employed. Practicing PTs and PT students were recruited to fill out a self-administered questionnaire to rank nine different professions (including PT) across different dimensions. Further, participants reported on their job satisfaction and participated in a semistructured interview regarding their responses.

**Results:**

A total of 175 individuals participated in this study. The physical therapy profession was rated 6^th^ on level of education, last (9^th^) on level of income and level of social standing, 5^th^ on level of responsibility, and 3^rd^ on level of usefulness. In the overall occupational prestige, the physical therapy profession was ranked the lowest compared to the other professions. Lastly, the data from the semistructured interviews corroborated the findings identified from the quantitative aspect of our study. *Discussion*. The overall results of the study indicate that the current perceived level of occupational prestige among Saudi PTs and PT students is somewhat disappointing. Participants generally viewed their profession in a low occupational prestige status, which is in contrast to previous studies conducted in other countries. While some of these results can be explained by the nature and history of the profession in Saudi Arabia, Academic institutions and policy makers should make an effort to promote the occupational prestige of the physical therapy profession.

## 1. Introduction

Physical therapists (PTs) are essential members of the healthcare team [1–3]. They evaluate and treat individuals of all ages who have a variety of injuries or disabilities or other health conditions that require their care [2, 4]. PTs can also provide consultation to individuals who want to be healthier and avoid future problems or injuries [2, 4]. PTs may also specialize in specific fields such as pediatrics, neurology, or sports to help individuals overcome physical impairment and dependency [5].

The need for PTs continues to grow, especially with the increase of the disabled population, individuals with limited function, and prolonged life expectancy [2, 5, 6]. The World Health Organization (WHO) estimates that about 1 billion people live with some form of disability [7]. Further, the WHO estimates that between the years 2000 and 2050, the world population aged over 65 years will triple from 600 million to 2 billion [6]. In the Kingdom of Saudi Arabia (KSA), studies show that disability is one of the most imperative social and economic burdens of the Ministry of Health in KSA [8]. It is estimated that 3.73% of the population suffer some form of functional disability that limit their function and independence [8]. All of the previous figures highlight the importance of PTs and the continuous need for their services and skills.

A key feature of any occupation is that it holds a certain social status relative to other occupations. In modern societies, social status is largely determined by an individual's occupation or the occupation of their parents rather than an inherited social standing [9]. Further, evidence suggests that the individual's character, level of intelligence and education, ability, and personal acceptability are assumed based on their occupation [9, 10]. There are certain occupations that are commonly associated with power, prestige, and material reward such as medicine and legal professions [9]. The social standing of an occupation is often referred to as occupational prestige [11]. Occupational prestige is often evaluated as how that particular occupation is regarded relative to other occupations by different groups in the society [11]. Occupational prestige is a useful indicator of a profession's marketability and desirability [11].

The social status of an occupation is often important to its members and to their job satisfaction, and physical therapy (PT) is no exception [12]. Previous studies that reported on the occupational prestige of PT had inconsistent findings. Some studies reported that medical practitioners and laymen regarded PT with low social standing, while other studies reported that PT has a high social standing [13–16]. In other studies evaluating occupational prestige among PT students, their results showed that students regarded PT as an intermediate occupation rather than a profession [11], while in another study, PT students ranked their future profession as having an overall high prestige alongside a doctor and a judge [12].

Only a few studies have investigated PT's perceptions regarding their occupational prestige and job satisfaction [12]. Further, in KSA, there are no studies investigating these concepts. It is important to maintain desirability for studying and practicing PT, especially with the predicted increase in populations who require PT services. Thus, the purpose of the current study is to determine Saudi PT's (students and graduates) perception of their occupational prestige and satisfaction with their career choice.

## 2. Methods

### 2.1. Study Design

A cross-sectional study design with a concurrent mixed-method approach was conducted. For the quantitative part of the study, participants completed a self-reported questionnaire. For the qualitative part, participants underwent an interview process, which is clarified below. Ethical approval was obtained prior to commencing the study from the local institutional bioethics committee (1442-189-315). All participants provided a written informed consent.

### 2.2. Recruitment and Setting

Participants were recruited using advertisements, word of mouth, and through local and national PT departments and centers. Further, PT students in multiple KSA universities were recruited through the same process. The advertisement indicated that a study is under process and PTs or student PTs are needed for the study. The questionnaire process was performed either in-person or through an online meeting software.

### 2.3. Participants

For the study, we aimed to recruit a convenience sample (~150-200 participants) from various sites within KSA in order to ascertain the diversity of PTs and PT students. Participants were included in the study if they were either (i) a practicing and licensed Saudi PT who works in a full-time position either in the public or private sector or (ii) a PT student who is currently studying full time in a PT program and beyond his/her 2nd year of the program.

Once a potential participant was identified and provided a written informed consent, the following information was collected for screening purposes. These were collected prior to any further contact or procedures and included date of birth, sex, years of experience, years since graduation, and place of work (for PTs) and years in PT program and university name (for PT students).

### 2.4. Procedures and Outcomes

All participants completed a questionnaire adapted from the study by Turner (2001). The questionnaire involves rating various professions across five dimensions. For our study, the professions were PT, doctor, pharmacist, nurse, an army or police officer, school teacher, lawyer, university professor, and engineer. The five dimensions are levels of education, income, social standing, responsibility, and usefulness to the society. Participants were asked to rate these professions on a 6-point bipolar interval scale on the aforementioned five dimensions. On the scale, 1 and 2 represent low, 3 and 4 moderate, and 5 and 6 high ranking. To prevent possible order effects, four versions of the questionnaires were used, two orders for the professions (first order and reverse) and two orders for the dimensions (first order and reverse). Additionally, in order to assess job satisfaction, participants were asked to rate their overall job satisfaction on a scale from 1 (representing low satisfaction) to 10 (representing high satisfaction). Job satisfaction question was only administered with PTs and not with PT students.

For the qualitative part of the study, semistructured interviews were conducted. A stratum of the participants was asked to elaborate on their choices regarding how they rated the PT occupation on the previous five dimensions. The question will be “why did you rate the PT profession as X on the 1-6 scale for that dimension?” All interviews were digitally recorded and transcribed verbatim. The interviewer also collected additional notes during this process.

### 2.5. Data Analysis

Data was summarised using means and standard deviations and frequencies. Further, the overall occupational prestige for each profession was determined by summation of all dimensions. A repeated measures analysis of variance (ANOVA) was performed separately on each of the dimensions. For each analysis of variance, the within-subject factor was the 9 professions. Three different sets of ANOVA were performed; the first was for all participants; the second were for PTs only; and the third set of ANOVAs was for PT students. The between-subject factors for all participants were gender, age, position (i.e., PTs or PT students) and order. The between-subject factors for PTs were gender, age, years of experience, and work site (i.e., private or government). The between-subject factors for PT students were gender, age, university and years in program. Given the extent of multiple statistical analyses involved in the study, the *p* value was set at 0.01.

The level of agreement among participants for each dimension was assessed using Kendall's coefficient of concordance (Kendall's *W*). In addition, a nonparametric multidimensional scaling analysis was performed to assess overall prestige.

For the qualitative data, a thematic analysis was performed on the transcribed interviews and the notes collected during interviews. The researcher utilized an inductive approach for analysis, gathering insights without preconceived or existing theories or categories, and followed the 6 phases of thematic analysis. Researcher made notes of the interrelationships, connections, and patterns that emerged from the data to identify themes and subtopics [17].

## 3. Results

The results of the current study showed that 175 individuals participated in the study's questionnaire. Of those 175 participants, 27 individuals underwent the semistructured interviews for the qualitative segment of the study. The majority of participants were PT students (*n* = 122) compared to PTs (*n* = 53). Similarly, the majority of participants were females (*n* = 109) compared to males (*n* = 66). The average age for PTs was 26.7 ± 4.2, and the average age for PT students was 23.5 ± 2.3.  Table 1 shows the summary of participants' characteristics.

When analysing the data for all participants, results for each of the ANOVA showed that the within-subject factor (the different professions) was highly significant (*p* < 0.0001). Thus, the professions were differentiated on each of the five dimensions. The analyses for between-subject factors revealed age effects on all five dimensions. Further, position effects were found for level of social standing dimension; and gender effects were found for level of responsibility dimension. No other significant effects were found.

When analysing the data for PTs only, results for each of the ANOVA showed that the within-subject factor was highly significant (*p* < 0.0001). Similar to the above, the professions were differentiated on each of the five dimensions. The analyses for between-subject factors revealed years of experience effects on three dimensions (levels of education, income, and social standing). Further, age and gender effects were found for level of social standing dimension. No other significant effects were found.

When analysing the data for PT students only, results for each of the ANOVA showed that the within-subject factor was highly significant (*p* < 0.0001). Similar to the previous two sets of ANOVA, the professions were differentiated on each of the five dimensions. The analyses for between-subject factors revealed university effects for levels of education, income, and responsibility. Further, age effects were significant for levels of income and usefulness; and years in program effects were significant for level of responsibility. No other significant effects were found.

The level of agreement (Kendall's *W*) was greater among PTs for two dimensions (levels of income and responsibility), while greater among PT students for the remaining three dimensions (Table 2). Level of income has the highest degree of consensus across all five dimensions, while level of usefulness had the lowest degree of consensus.

### 3.1. Level of Education

**Figure 1:**
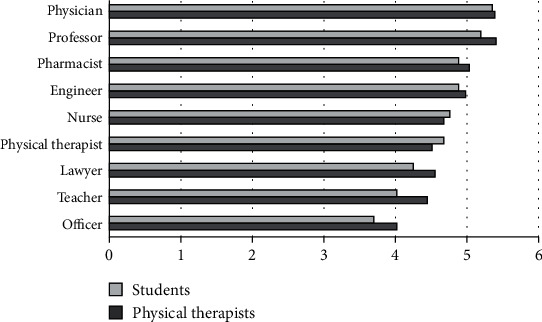
Mean ranks for level of education.

The perceived level of education for the different professions showed that PTs and PT students placed the physician and the university professor at the highest level, while the army or police officer and school teacher were placed at the lowest level (Figure 1). The physical therapy profession was ranked 7^th^ by PTs and 6^th^ by PT students.

### 3.2. Level of Income

**Figure 2:**
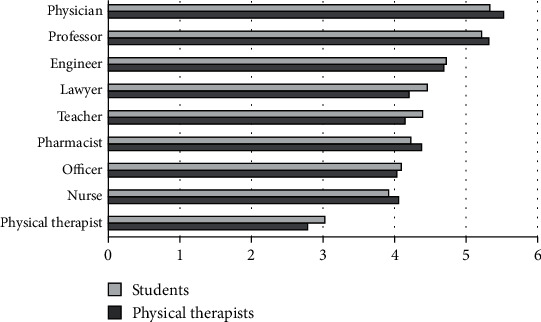
Mean ranks for level of income.

The perceived level of income for the different professions showed that PTs and PT students placed the physician and the university professor at the highest level, while the physical therapist, nurse, and army or police officer were placed at the lowest level (Figure 2).

### 3.3. Level of Social Standing

**Figure 3:**
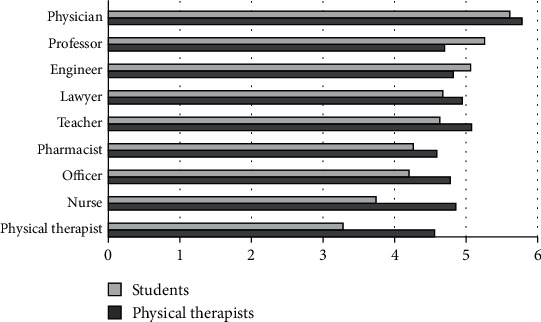
Mean ranks for level of social standing.

The perceived level of social standing for the different professions showed that PTs and PT students placed the physician at the highest level, followed by army or police officer as ranked by PTs or followed by university professor as ranked by PT students. For the lowest ranked professions, PTs ranked the physical therapy profession and the school teacher as the lowest, while PT students ranked the physical therapy profession and the nurse as the lowest (Figure 3).

### 3.4. Level of Responsibility

**Figure 4:**
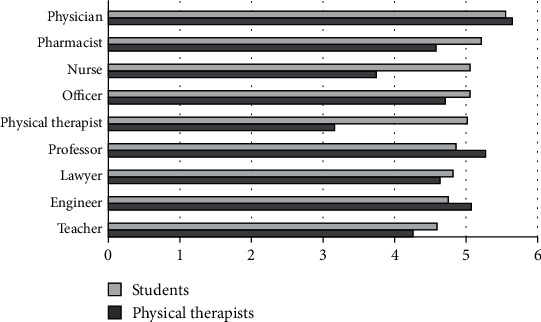
Mean ranks for level of responsibility.

The perceived level of responsibility for the different professions showed that PTs and PT students placed the physician at the highest level, followed by the university professor as ranked by PTs or followed by the pharmacist as ranked by PT students. For the lowest ranked professions, PTs ranked the physical therapy profession and the nurse as the lowest, while PT students ranked teachers and engineers as the lowest (Figure 4). Further, PT students ranked the physical therapy profession 5^th^ compared to other professions.

### 3.5. Level of Usefulness

**Figure 5:**
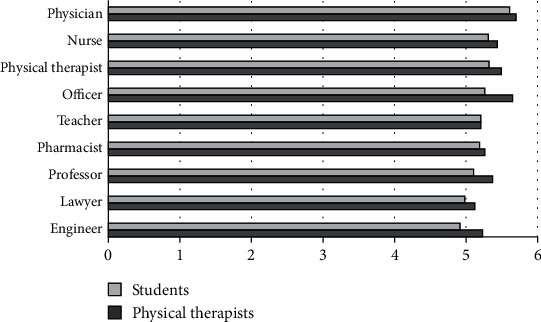
Mean ranks for level of usefulness.

The perceived level of usefulness for the different professions showed that PTs and PT students placed the physician at the highest level, followed by the army or police officer as ranked by PTs or followed by the nurse as ranked by PT students. For the lowest ranked professions, PTs ranked the lawyer and school teacher as the lowest, while PT students ranked engineers and lawyers as the lowest (Figure 5). The physical therapy profession was ranked 3^rd^ by both PTs and PT students.

### 3.6. Combined Dimensions

**Figure 6:**
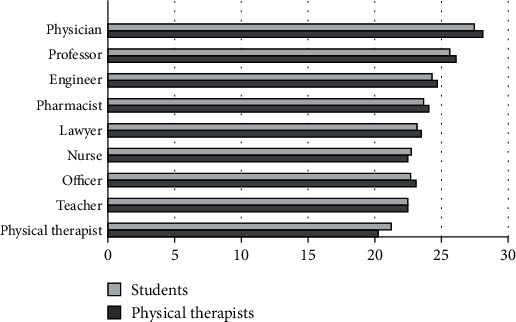
Mean ranks for overall occupational prestige.

**Figure 7:**
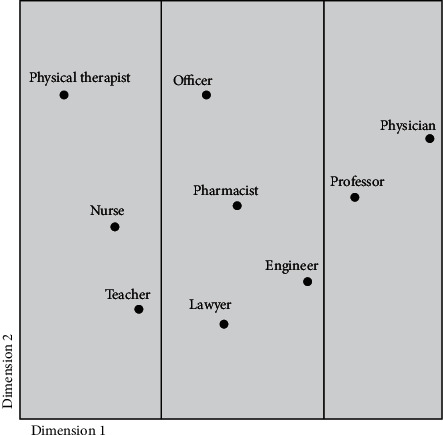
Euclidean model for overall occupational prestige.

The overall occupational prestige for all dimensions showed that PTs and PT students placed the physician and the university professor at the highest level, while the physical therapist profession and the school teacher were ranked the lowest (Figure 6). The results of the multidimensional scaling analysis are shown in (Figure 7). Within the space, the occupations can be divided into three groups, from high occupational prestige at one side (physician and professor) to low occupational prestige standing at the other extreme (physical therapist, nurse, and teacher).

### 3.7. Qualitative Data

Table 3 provides examples of excerpts taken from individuals who participated in the semistructured interview. For level of education, it was apparent from participants' responses that the level of education for PTs is sufficient for practicing the profession. However, physical therapy—as other medical professions—involves continuous education and training. The cost of educational courses is often high, which can be challenging for PTs. Another point reported by participants was that most PT centers require therapists to work with a variety of cases and do not promote PT specialities (i.e., orthopedic, neurology).

For level of income, the common theme is that income is low and not proportionate to the amount of work performed by PTs, which can be disappointing. While some participants did report being content with their current income, it was apparent that the vast majority were not satisfied with it, especially those working in the private sector.

For level of social standing, a common theme emerged from the participants' responses as they reported that PTs are often viewed with a low social standing. In fact, the majority of participants reported that PTs are often wrongfully viewed by the community as massage specialists and not regarded highly as other medical professionals.

For level of responsibility, the majority of participants' responses indicated that the level of responsibility for PTs is enormous. PTs often deal with individuals with disabilities and those who are at risk of injury. Therefore, educational institutions have an obligation towards PT students to prepare them to be able to take on this responsibility. However, some of the PTs who participated in the semistructured interviews noted that the level of responsibility (after they have practiced their profession) is lower than what they expected (or taught) during their university years.

For level of usefulness, two (contradicting) themes emerged from participants' responses in this prat of the interview. For PT students, a few of them reported that PT is important to the society; however, it is not an essential part of healthcare. On the other hand, PTs unanimously reported that PTs are essential and useful to the society. In fact, they are an integral part of the healthcare system.

## 4. Discussion

The present study is aimed at determining the perceived level of occupational prestige and satisfaction with career choice among Saudi PTs and PT students. The overall results of the study indicate that the current perceived level of occupational prestige among the participants is somewhat disappointing. It appears that physical therapy as a profession is generally perceived as low standing across different dimensions. These findings are in contrast to previous similar studies investigated across different countries [11, 12, 18, 19].

Participants in the current study ranked the physical therapy profession as 7^th^ (PTs) and 6^th^ (PT students). This finding is in contrast to previous studies as the physical therapy profession was generally rated higher compared to other professions [11, 12, 18, 19]. For level of income, our participants ranked the physical therapy profession at the lowest level. In comparison, previous studies conducted in other countries (Australia, United Kingdom, United States, and Nigeria) reported that the physical therapy profession was generally rated intermediate compared to other professions [11, 12, 18, 19]. Similarly, on the level of social standing, participants ranked the physical therapy profession at the lowest level. In previous studies, the physical therapy profession was generally ranked intermediate [11, 12, 18, 19].

For level of responsibility, the participants ranked the physical therapy profession 5^th^ compared to other professions. This finding is similar to previous studies, as the physical therapy profession was previously ranked 6^th^ [11], 3^rd^ [12], 4^th^ [19], and 5^th^ [18]. Similarly, for level of usefulness, participants ranked the physical therapy profession as 3^rd^ compared to other professions. This finding corroborates previous studies, as the physical therapy profession was previously ranked 5^th^ [11], 3^rd^ [12], 2^nd^ [19], and 3^rd^ [18]. For the overall occupational prestige, the participants in our study viewed the physical therapy profession as having the lowest occupational prestige compared to the other professions. This was also corroborated by the Euclidean space. However, compared to previous studies, the physical therapy profession is generally viewed to have an intermediate occupational prestige [11, 18, 19] or a high occupational prestige [12].

In regard to job satisfaction, our participants generally rated their job satisfaction as low (4/10). Previous studies investigating job satisfaction among Saudi PTs have reported similar results [20, 21]. In study published in 2015, the authors reported that job satisfaction reported by Saudi PTs was 37%. Further, the authors reported that gender, age, relationship with supervisors and managers, and work environment were significant predictors of job satisfaction. It was found that female PTs generally had higher levels of job satisfaction compared to male PTs [20]. In a more recent study that investigated job satisfaction among Saudi PTs with emphasis on leadership style, the authors reported similar results. It was found that the majority of participants were neither fully satisfied nor fully dissatisfied with their job. Similar to the previous study, it was found that female PTs were generally more satisfied than male PTs [21]. In the current study, the majority of participants were females (66 males/109 females). However, job satisfaction was still found to be low. More studies are needed to examine the predictors for job satisfaction among Saudi PTs and discern the differences between genders in relation to job satisfaction.

The qualitative findings of our study were congruent with the quantitative aspect. Participants elaborated on their choices regarding the occupational prestige of the physical therapy profession. Concerns regarding the level of income and level of social standing were the most prominent issues raised by the respondents. The aforementioned findings related to the quantitative and qualitative aspects of the study may be explained by the nature and history of the physical therapy profession in Saudi Arabia.

The physical therapy educational programs in Saudi Arabia were introduced somewhat later compared to other countries [22]. Furthermore, the Saudi physical therapy association (SPTA), which is the scientific association for the profession was founded only 20 years ago (2002). As such, the physical therapy profession in Saudi Arabia is still in development and lacks the strong and substantiated heritage that is observed for the profession in other countries. In a study conducted to assess the awareness, perceptions, and beliefs of physicians in Saudi Arabia regarding PTs, the majority of respondents reported that they have a negative perception about physical therapy and a large proportion of the respondents did not entirely understand the roles and responsibilities of PTs [1]. As mentioned above, the need for PTs will continue to grow due to the increasing number of people with disabilities and impairments and the increasing number of older adults who require PT care. Therefore, the challenges facing PTs highlighted within the current study should be addressed in order to maintain and increase the desirability of the physical therapy profession and maintain an adequate number of people practicing PT.

To our knowledge, this is the first study that examined the occupational prestige among Saudi PTs and PT students. However, the current study is not without limitations. The main limitation of the current study is that it is sample-specific (Saudi PTs and Pt students) and cannot be widely generalized. Second, the sample size recruited for the study was somewhat small compared to other similar studies. However, the recruited participants were from a wide variety of universities and regions within Saudi Arabia, thus, representing a broad sample from the target population.

## 5. Conclusion

PTs and PT students in Saudi Arabia do not perceive their profession as highly as their counterparts in other countries. Academic institutions, scientific associations, and policy makers should make an effort to promote the current occupational status of the physical therapy profession in Saudi Arabia and, thus, guarantee that potential PTs keep enrolling in PT educational programs to fill the need for the profession, especially with the continuous and future demand for profession in Saudi Arabia.

## Figures and Tables

**Table 1:** Participants' characteristics.

Demographics	Physical therapists	Physical therapy students
*N* (males/females)	53 (23/30)	122 (43/79)
Age: mean (SD)	26.7 years (4.2)	23.5 years (2.3)
Years of experience: mean (SD)	3.4 years (5.4)	
Years in university: median (range)		5 years (3-7)
Job satisfaction: median (range)	4/10 (1-9)	

**Table 2:** Degree of consensus (Kendall's coefficient of concordance—*W*).

Dimensions	Physical therapists	Physical therapy students	All participants
Level of education	0.253	0.273	0.259
Level of income	0.397	0.342	0.357
Level of social standing	0.121	0.370	0.249
Level of responsibility	0.267	0.114	0.106
Level of usefulness	0.089	0.089	0.082
All dimensions	0.245	0.277	0.236

**Table 3:** Excerpts taken from participants' semi-structured interviews.

Items	Excerpts from participants
Level of education	“Education for PTs is a continuous process, it never ends. The university education only lays out the foundations for you, but it is up to you to build up your knowledge” *P11*
Level of income	“A physical therapist income in Saudi Arabia is low, and not proportionate at all with the amount of time and effort performed by a PT” *P23*
Level of social standing	“Most of my patients often think that I am a massage therapist and do not understand what is my job and how I can help them” *P23*
Level of responsibility	“We have a big responsibility towards our patients. I worry about my patients constantly, especially those who are at risk of falls” *P5*
Level of usefulness	“In my field of practice -pediatrics- my mission is to help children with disabilities overcome any challenges they may have; and to assure parents or guardians that their children -who have a disability- should live their life to its fullest potential” *P19*

## Data Availability

The data used to support the findings of this study are available from the corresponding author upon request.
